# Development and validation of grade‐based prediction models for postoperative morbidity in gastric cancer resection using a Japanese web‐based nationwide registry

**DOI:** 10.1002/ags3.12269

**Published:** 2019-06-20

**Authors:** Yoshio Haga, Hiroaki Miyata, Akira Tsuburaya, Mitsukazu Gotoh, Kazuhiro Yoshida, Hiroyuki Konno, Yasuyuki Seto, Yoshiyuki Fujiwara, Hideo Baba

**Affiliations:** ^1^ Japan Community Healthcare Organization Amakusa Central General Hospital Amakusa‐shi Japan; ^2^ Database Committee The Japanese Society of Gastroenterological Surgery Tokyo Japan; ^3^ Department of Surgery Ozawa Hospital Odawara Japan; ^4^ Department of Surgical Oncology Gifu University Graduate School of Medicine Gifu Japan; ^5^ Hamamatsu University School of Medicine Hamamatsu Japan; ^6^ The Japanese Society of Gastroenterological Surgery Tokyo Japan; ^7^ Division of Surgical Oncology Department of Surgery School of Medicine Tottori University Faculty of Medicine Yonago Japan; ^8^ Department of Gastroenterological Surgery Graduate School of Medical Sciences Kumamoto University Kumamoto Japan

**Keywords:** gastric cancer, morbidity, National Clinical Database, risk model, surgery

## Abstract

**Aim:**

Gastric cancer is the second leading cause of cancer death worldwide. Surgery is the mainstay treatment for gastric cancer. There are no prediction models that examine the severity of postoperative morbidity. Herein, we constructed prediction models that analyze the risk for postoperative morbidity based on severity.

**Methods:**

Perioperative data were retrieved from the National Clinical Database in patients who underwent elective gastric cancer resection between 2011 and 2012 in Japan. Severity of postoperative complications was determined by Clavien‐Dindo classification. Patients were randomly divided into two groups, the development set and the validation set. Logistic regression analysis was used to build prediction models. Calibration powers of the models were assessed by a calibration plot in which linearity between the observed and predicted event rates in 10 risk bands was assessed by the Pearson *R* ^2^ statistic.

**Results:**

We obtained 154 278 patients for the analysis. Prediction models were constructed for grade ≥2, grade ≥3, grade ≥4, and grade 5 in the development set (n = 77 423). Calibration plots of these models showed significant linearity in the validation set (n = 76 855): *R* ^2^ = 0.995 for grade ≥2, *R* ^2^ = 0.997 for grade ≥3, *R* ^2^ = 0.998 for grade ≥4, and *R* ^2^ = 0.997 for grade 5 (all: *P *<* *0.001).

**Conclusion:**

Prediction models for postoperative morbidity based on grade will provide a comprehensive risk of surgery. These models may be useful for informed consent and surgical decision‐making.

## 1 INTRODUCTION

Gastric cancer is the fourth leading cause of cancer incidence and second leading cause of cancer death worldwide.^1^ Surgery is the only treatment that provides a chance for a cure against gastric cancer except for endoscopically resectable early tumors.^2^ Surgery for gastric cancer can be safely carried out in most cases. Thirty‐day postoperative mortality rates of gastric cancer resections were <1% according to a national database in Japan.^3^, ^4^ Nevertheless, elderly patients who require gastric cancer resections are increasing with an aging society in developed countries. Some of these patients have multiple comorbidity conditions, such as hypertension, diabetes mellitus, old myocardial infarction, and old brain infarction, and patients sometimes go back and forth between nursing homes and hospitals. These patients have limited reserve capacity and sometimes suffer from postoperative complications. Therefore, prediction of postoperative morbidity is still important.

There are several prediction models of postoperative morbidity for gastric cancer patients.^5^, ^6^, ^7^, ^8^, ^9^, ^10^ Nevertheless, patients may be uncertain about how much damage they will sustain because there are no prediction models based on severity. For example, patients can eat meals and function normally because of a wound infection. By contrast, patients are kept in bed with various tubes because of an abdominal abscess. To share information on postoperative morbidity, prediction of grade‐specific morbidity rates is needed for both patients and doctors.

The National Clinical Database (NCD) in Japan was developed in collaboration with the National Surgical Quality Improvement Program (NSQIP) in USA with a shared goal of creating a standardized surgery database for quality improvement. NCD and NSQIP have developed systems using standardized variable definitions to collect data on risk factors and outcomes after surgery.^11^ These databases collect prospective rather than retrospective data. Because patient registration for the board certification system by the Japan Surgical Society can be carried out only by the NCD system, the NCD now covers more than 97% of total surgical procedures in Japan.^12^ This study was undertaken to construct prediction models to estimate grade‐specific postoperative morbidity in gastric cancer resection using large NCD data.

## 2 METHODS

### 2.1 Study design

This study was prepared in response to a public call for research using NCD data by the Japanese Gastric Cancer Association (JGCA) in 2014. The study protocol was approved by the Council of JGCA on June 3, 2014. This was a retrospective analysis of data from NCD data.

### 2.2 Patients

Patients were selected who underwent partial gastrectomy, total gastrectomy, total gastrectomy with splenectomy, and total gastrectomy with distal pancreatectomy and splenectomy in combination with the main disease of gastric cancer between 2011 and 2012. Exclusion criteria were emergency operations, concomitant cancer, preoperative systemic inflammatory response syndrome (SIRS), preoperative sepsis, and recurrent disease because standard operations are usually avoided under these conditions.

### 2.3 Data collection

Thirty‐eight preoperative conditions including comorbidities, past history, and functional status were collected with 17 preoperative laboratory data. Sixteen intraoperative data were also collected including type of surgery, American Society of Anesthesiologists Physical Status (ASA‐PS), operative duration, blood loss, intraoperative problems, and information of metastatic organs.^11^ The outcome of this study was postoperative morbidity according to the Clavien‐Dindo classification.^13^ Postoperative complications were defined as adverse events that occurred within 30 days after operation. Absence or presence of 47 complications was recorded with Clavien‐Dindo grades. Other complications were also recorded with their name and Clavien‐Dindo grade.

### 2.4 Statistical analysis

Patients were divided into two groups, a development set and a validation set, by a random sampling method. Univariate analysis of each predictor was conducted using chi‐squared tests with Yates correction when appropriate. Using the significant variables by univariate analysis, a stepwise increase logistic regression analysis was carried out to construct a prediction model for graded postoperative morbidity. When entering the variables into the multivariate analyses, we excluded the variables with ambiguous definitions, such as “transport by ambulance.” We also excluded the variables with an incidence less than 1%, such as “ventilator dependent.” We further excluded the variables with odds ratios that were close to 1 even though they were significant. When the variables were closely related to each other, such as hemoglobin levels and hematocrit, we excluded the variable with the smaller odds ratio.

Discriminative and calibration power of the model was carried out using area under receiver‐operating characteristics curve (AUC) and a calibration plot, respectively. In the calibration plot, patients were divided into 10 risk bands according to the predicted event rates. Each risk band was set to have an equal number of patients. Linearity between the observed and predicted event rates was assessed by Pearson's *R* ^2^ statistic.

## 3 RESULTS

We obtained 154 278 patient datasets for analysis and divided them into two groups, a development set and a validation set. Demographic data are shown in Table 1. Both groups were well balanced in preoperative factors, intraoperative factors, and postoperative morbidity.

**Table 1:** Demographic data of patients who underwent elective gastric cancer resection between 2011 and 2012 in Japan

	Development set (n = 77 423)	Validation set (n = 76 855)
Preoperative factors		
Age, y, mean (SD)	69.3 (11.3)	69.2 (11.3)
Male	53 529 (69.1%)	53 056 (69.0%)
Diabetes mellitus	12 659 (16.4%)	12 366 (16.1%)
COPD	3032 (3.9%)	3067 (4.0%)
Cerebrovascular disease	2958 (3.8%)	2890 (3.8%)
Previous PCI	2002 (2.6%)	1959 (2.5%)
Previous cardiac surgery	906 (1.2%)	900 (1.2%)
Previous PVD surgery	407 (0.5%)	443 (0.6%)
Bleeding disorder	2709 (3.5%)	2676 (3.5%)
Weight loss ≥10%	4445 (5.7%)	4474 (5.8%)
Respiratory distress, any	1598 (2.1%)	1519 (2.0%)
ADL, any assistance	3295 (4.3%)	3303 (4.3%)
Ascites	1065 (1.4%)	1096 (1.4%)
BMI, median (IQR)	22.0 (19.8‐24.2)	22.0 (19.8‐24.2)
ASA‐PS ≥3	7577 (9.8%)	7366 (9.6%)
Disseminated disease	1348 (1.7%)	1276 (1.7%)
Laboratory data		
WBC >11 000/μL	1493 (1.9%)	1482 (1.9%)
Platelet <80 000/μL	384 (0.5%)	363 (0.5%)
Albumin <4.0 g/dL	27 811 (35.9%)	27 310 (35.5%)
Na <135 mmol/L	2357 (3.0%)	2236 (2.9%)
Creatinine >1.2 mg/dL	5356 (6.9%)	5135 (6.7%)
AST >35 IU/mL	6009 (7.8%)	5950 (7.7%)
ALP >600 IU/mL	467 (0.6%)	481 (0.6%)
CRP >1.0 mg/dL	6573 (8.5%)	6428 (8.4%)
Prothrombin time‐INR >1.25	1866 (2.4%)	1928 (2.5%)
Intraoperative factors		
Blood loss, g, median (IQR)	190 (70‐400)	194 (70‐405)
Operation time, min, median (IQR)	250 (196‐315)	251 (196‐315)
Type of gastrectomy		
Partial gastrectomy without LN dissection	4577 (5.9%)	4643 (6.0%)
Partial gastrectomy	46 411 (59.9%)	45 913 (59.7%)
Total gastrectomy	23 832 (30.8%)	23 735 (30.9)
Proximal gastrectomy	647 (0.8%)	673 (0.9%)
Total gastrectomy with splenectomy	2277 (2.9%)	2207 (2.9%)
Total gastrectomy with PS	327 (0.4%)	358 (0.5%)
Postoperative morbidity		
Grade 2	12 806 (16.5%)	12 828 (16.7%)
Grade 3	6006 (7.8%)	6046 (7.9%)
Grade 4	1604 (2.1%)	1552 (2.1%)
Grade 5	1069 (1.4%)	1031 (1.3%)

ADL, activities of daily living; ALP, alkaline phosphatase; ASA‐PS, American Society of Anesthesiologists Physical Status; AST, aspartate aminotransferase; BMI, body mass index; COPD, chronic obstructive pulmonary disease; CRP, C‐reactive protein; INR, international normalized ratio; IQR, interquartile range; LN, lymph node; PCI, percutaneous coronary intervention; PS, pancreatosplenectomy; PVD, peripheral vascular disease; WBC, white blood cell count.

Table 2 shows the parameters and coefficients of the grade‐specific models for postoperative morbidity that were generated in the development set. The predicted event rate (*p*) for each outcome was calculated as follows: ln[*p*/(1 − *p*)] = β_0_ + ∑β_i_ *X*_*i*_, where β_0_ is a constant, β_i_ is a coefficient, and *X*_*i*_ is a variable.

**Table 2:** Coefficients of logistic regression models for postoperative morbidity

Variables	Grade ≥2	Grade ≥3	Grade ≥4	Grade 5
Age, y	—	—	0.244	0.291
Distal gastrectomy without LN dissection	—	—	—	0.231
With colectomy	0.568	0.541	—	—
Total or proximal gastrectomy	0.413	0.463	0.580	0.566
With splenectomy	0.523	0.413	—	—
Male	—	0.460	0.330	0.199
Respiratory distress, any	0.420	—	0.474	0.670
Weight loss ≥10%	0.271	0.286	0.431	0.534
Previous PCI	0.314	0.256	0.377	0.305
Previous cardiac surgery	0.253	—	—	—
Previous PVD surgery	0.314	—	—	—
ADL, any assistance	0.515	0.612	0.715	0.821
Cerebrovascular disease	0.403	0.292	0.509	0.412
Ascites, any	0.491	0.454	0.830	1.040
COPD	0.294	0.365	0.214	—
PVD	—	0.541	—	—
Disseminated disease	0.266	0.515	0.922	1.092
Bleeding disorder	0.561	—	0.766	0.691
ASA‐PS ≥4	0.819	0.535	0.723	—
ASA‐PS ≥3	0.360	0.336	0.410	0.423
BMI >25	—	—	0.203	—
BMI >26	0.287	—	—	—
WBC >11 000/μL	0.261	—	0.670	0.691
Platelets <80 000/μL	0.596	0.782	0.803	0.879
Albumin <4.0 g/dL	0.294	0.276	—	—
Albumin <3.8 g/dL	—	—	0.446	0.624
Na <138 mmol/L	—	—	0.379	0.463
Na <135 mmol/L	0.298	—	—	—
Creatinine >2.0 mg/dL	—	—	0.500	—
Creatinine >1.2 mg/dL	—	0.282	0.518	—
AST >35 IU/mL	—	—	0.290	0.341
ALP >600 IU/mL	—	—	0.781	0.868
CRP >1.0 mg/dL	—	—	0.276	0.322
Prothrombin time‐INR >1.25	—	—	0.245	0.364
Constant	−2.441	−3.337	−6.094	−6.880

ADL, activities of daily living; ALP, alkaline phosphatase; ASA‐PS, American Society of Anesthesiologists Physical Status classification; AST, aspartate aminotransferase; BMI, body mass index; COPD, chronic obstructive pulmonary disease; CRP, C‐reactive protein; INR, international normalized ratio; PCI, percutaneous coronary intervention; PVD, peripheral vascular disease; WBC, white blood cell count.

(Predicted event rate (*p*) for each outcome was calculated as follows: ln [*p*/(1 − *p*)] = β_0_ + ∑β_*i*_ *X*_*i*_, where β_0_ is a constant, β_*i*_ is a coefficient, and *X*_i_ is a variable).

"–" means no coefficients.

**Figure 1:**
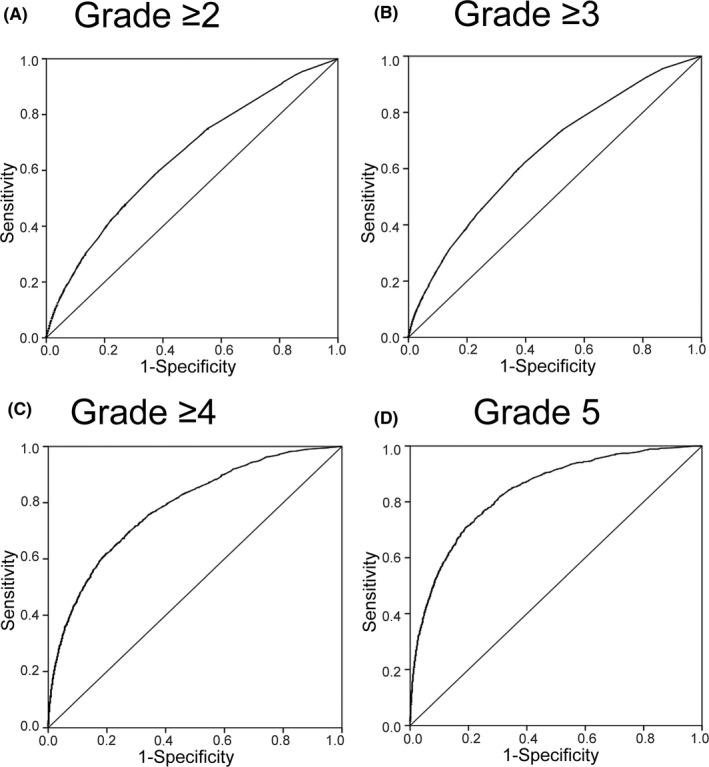
Discriminative power of prediction models for postoperative morbidity in gastric cancer resection. Receiver operating characteristic curve analysis for each prediction model was carried out in a validation set (n = 76 855)

Figure 1 shows the AUC of models in the validation set. When dependent variables included lower grades of complications, the AUC became lower. The AUC (95% CI) were 0.656 (0.650‐0.661) for grade ≥2, 0.668 (0.661‐0.675) for grade ≥3, 0.794 (0.783‐0.806) for grade ≥4, and 0.839 (0.828‐0.851) for grade 5.

**Figure 2:**
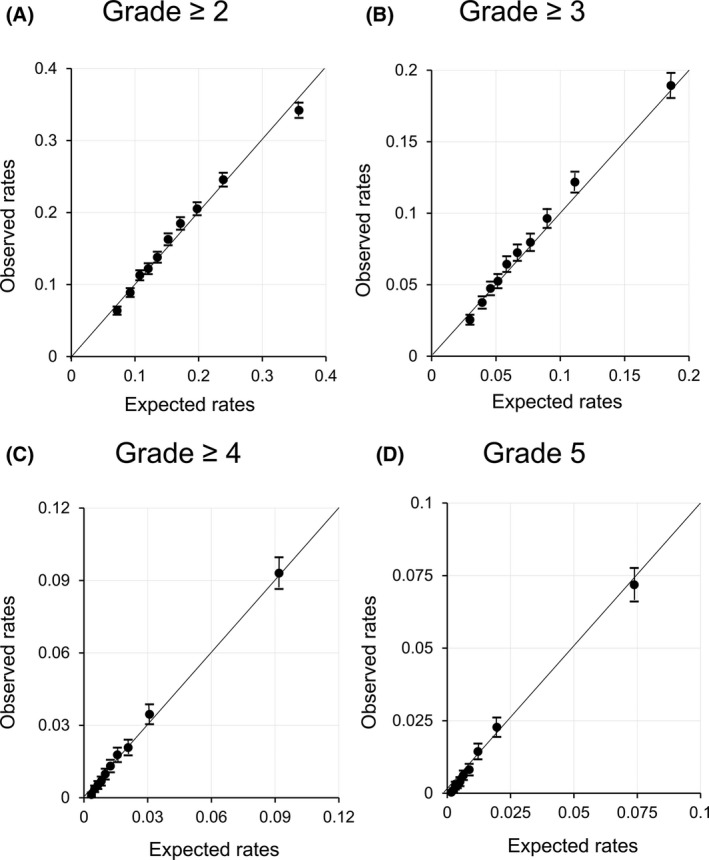
Calibration plot of prediction models for postoperative morbidity in gastric cancer resection. Observed event rates (95% CI) were plotted with predicted event rates in 10 risk bands for each prediction model in the validation set (n = 76 855)

Figure 2 shows calibration plots of the models in the validation set. All the models fit well from the lower to the higher risk groups. These models showed a significant linearity between the observed and predicted event rates in 10 risk bands: *R* ^2^ = 0.995 for grade ≥2, *R* ^2^ = 0.997 for grade ≥3, *R* ^2^ = 0.998 for grade ≥4, and *R* ^2^ = 0.997 for grade 5 (all: *P *<* *0.001). Tables 3 and 4 show the observed event rates in each risk band determined by the prediction models for grade ≥2 and grade ≥3, respectively.

**Table 3:** Risk of postoperative morbidity of patients classified as grade ≥2

Risk band	Predicted event rates	Observed event rates (95% CI)
1	<8.5%	6.4% (5.8% to 7.0%)
2	8.5% to <10.0%	8.9% (8.3% to 9.5%)
3	10.0% to <11.4%	11.3% (10.6% to 12.0%)
4	11.4% to <12.7%	12.2% (11.5% to 13.0%)
5	12.7% to <14.3%	13.8% (13.0% to 14.6%)
6	14.3% to <16.2%	16.3% (15.5% to 17.1%)
7	16.2% to <18.3%	18.5% (17.6% to 19.4%)
8	18.3% to <21.5%	20.5% (19.6% to 21.4%)
9	21.5% to <27.0%	24.6% (23.6% to 25.5%)
10	≥27.0%	34.2% (33.2% to 35.3%)

**Table 4:** Risk of postoperative morbidity of patients classified as grade ≥3

Risk band	Predicted event rates	Observed event rates (95% CI)
1	<3.5%	2.6% (2.2% to 2.9%)
2	3.5% to <4.3%	3.8% (3.3% to 4.2%)
3	4.3% to <4.8%	4.8% (4.3% to 5.2%)
4	4.8% to <6.2%	5.3% (4.8% to 5.8%)
5	6.2% to <7.1%	6.4% (5.9% to 7.0%)
6	7.1% to <8.2%	7.3% (6.7% to 7.8%)
7	8.2% to <9.8%	8.0% (7.4% to 8.6%)
8	9.8% to <12.8%	9.6% (9.0% to 10.3%)
9	12.8% to <25.2%	12.2% (11.5% to 12.9%)
10	≥25.2%	18.9% (18.1% to 19.8%)

## 4 DISCUSSION

We constructed and validated prediction models for postoperative morbidity following gastric cancer resections according to severity using large‐scale national data. Several prediction models have been reported for individual postoperative complications. Kikuchi et al^14^ developed prediction models for surgical site infections, anastomotic leakage, pancreatic fistula, pneumonia, prolonged pneumonia, and renal failure following total gastrectomy. Tu et al^15^ developed a nomogram to predict anastomotic leakage after gastrectomy for gastric cancer. However, there are no prediction models to account for all postoperative complications following gastric cancer resections. To the best of our knowledge, this is the first report regarding a grade‐stratified morbidity prediction system in this area. Postoperative complications may decrease long‐term survival in various cancer surgeries.^16^ Prediction of postoperative morbidity is becoming more important when considering early and long‐term prognosis. This system will help clinicians and patients with surgical decision‐making.

Postoperative complications following major gastrointestinal surgeries are an important outcome for patients. Complications decrease quality of life, prolong hospital stay, and increase medical costs. Postoperative complications include surgical complications that relate to surgery, and non‐surgical complications that are not related to surgery. Surgical complications, such as anastomotic leakage, may result from technical problems in addition to patient factors such as malnourishment and diabetes.^14^, ^17^ Non‐surgical complications, such as pneumonia, may depend on the patient's physiological conditions.^14^, ^18^ For both surgical and non‐surgical complications, the balance between patient reserve capacity and the degree of surgical stress may play a key role in the genesis of postoperative complications.^19^ Therefore, we postulate that postoperative morbidity by severity can be predicted using physiological and tumor‐related variables. For surgical factors, such as blood loss and operational time, these factors are dependent on tumor status and patient characteristics. For example, advanced carcinoma in the upper portion with significant lymph node enlargement requires total gastrectomy with extended lymph node dissection, which results in a longer operational time and a larger blood loss. If the patient has a higher body mass index, operational time and blood loss will be further increased. Therefore, these surgical factors are thought to be intermediate variables in the multivariate analysis, which may lead to overadjustment bias.^20^ The current models were constructed by analyzing the variables of physiological variables, metastatic status, and type of surgery. In preliminary analyses, we also constructed prediction models by adding surgical factors. Nevertheless, the predictive power of models with surgical factors was similar to models without surgical factors. Therefore, we used models without surgical factors.

The model's discriminative power became lower when lower grade complications were included as dependent variables. This phenomenon may result from the difficulty of predicting diverse complications. For example, the etiology of surgical site infection and postoperative pneumonia may be different. A previous study on total gastrectomy showed that predicting surgical complications is more difficult than predicting non‐surgical complications.^17^ For complications of grade ≥4, impaired patient reserve capacity may be important, and our model can predict these complications more precisely. The discriminative power indicates how well the model distinguishes events and non‐events on an individual patient basis. Because the AUC of the model for grade ≥2 or grade ≥3 was lower, these predicted event rates should not be used for patients that need an operation. However, when we classified a group of patients using the system as shown in Tables 3 and 4, we can assume the risk of these patients. In contrast, the models for grade ≥4 and grade 5 showed good discriminative and calibration power. Therefore, we can directly use the predicted event rates for these patients. Using these models, physicians and patients can be informed of operation risks more specifically.

When using these models, the quality of care of hospitals should be considered because a volume outcome relationship has been reported for gastric cancer surgeries.^21^, ^22^, ^23^, ^24^ The present models provide an average risk in Japan. Before using the models, each hospital should calculate postoperative morbidity rates according to the Clavien‐Dindo grading and compare them with NCD data. If the rates are far from NCD data, the predicted event rates should be adjusted in parallel to the observed rates.

A limitation of the present study was that the present validation set was not truly an external subset. We must analyze the predictive power in a truly external set such as future data. A validation study outside Japan will also add generalizability. Furthermore, the present models include many independent variables, which may burden the working time for data input. However, these variables are already incorporated into the NSQIP/NCD system and will not increase the workload at participating hospitals. The authors will upload a computer file which can compute risk of postoperative morbidity using the current models.

In conclusion, we constructed prediction models for grade‐based postoperative morbidity using large national data. These models show a clear portrait of postoperative risk in which clinicians and patients can share the same information.

## DISCLOSURE

Funding: This work was supported by a grant from the Japanese Gastric Cancer Association.

Conflicts of Interest: Authors declare no conflicts of interest for this article.

Ethical Statement: This study used only anonymized data from the NCD system. Ethical Guidelines for Medical and Health Research Involving Human Subjects in Japan defines no ethical obligation on such a study.
